# Clinical and Genetic Characterization of Russian Patients with von Hippel–Lindau Syndrome

**DOI:** 10.3390/ijms27198804

**Published:** 2026-10-01

**Authors:** Dmitry S. Mikhaylenko, Natalya N. Vasserman, Natalya B. Kuryakova, Ekaterina B. Kuznetsova, Nina A. Gorban, Anna A. Stepanova, Olga A. Shchagina, Alexander V. Polyakov, Dmitry V. Zaletaev, Sergey I. Kutsev, Vladimir V. Strelnikov

**Affiliations:** 1 Research Centre for Medical Genetics, Moscow 115522, Russiaschagina@med-gen.ru (O.A.S.); zalnem@mail.ru (D.V.Z.); vstrel@list.ru (V.V.S.); 2 Department of Translational Medicine and Biotechnology, I.M. Sechenov First Moscow State Medical University ( Sechenov University), Moscow 119991, Russia; 3 A.F. Tsyb Medical Radiological Research Center—Branch of the National Medical Research Center of Radiology, Obninsk 249036, Russia

**Keywords:** von Hippel–Lindau syndrome, renal cancer, *VHL* gene, sequencing, germline variant

## Abstract

Von Hippel–Lindau syndrome (VHLS) is an autosomal dominant hereditary cancer disease caused by germline mutations in the *VHL* tumor suppressor gene and characterized by clinical heterogeneity and phenotype–genotype associations. We studied the *VHL* gene in 535 Russian patients referred for VHLS diagnostics using Sanger sequencing, multiplex ligase-dependent probe amplification, and, in some cases, high-throughput sequencing. A total of 177 causative *VHL* variants were identified, including 129 point mutations and 48 extended deletions. Missense variants were the most frequent (60.3%) and predominantly localized at codons 167, 88, and 78. Approximately 88% of the analyzed missense variants and in-frame indels led to changes directly at the HIF-α- and ELOC-binding sites of pVHL. Patients with causative *VHL* variants had CNS hemangioblastoma (69.7%), retinal hemangioblastomas (41.4%), clear-cell renal carcinoma (CCRC) (27.3%), renal cysts (22.2%), pancreatic cysts (20.2%), and pheochromocytoma (7.1%). The frequency of multiple pathological changes in targeted organs was significantly higher in patients with causative variants than in the group without mutations, at 78.8% versus 21.1% (*p* < 0.0001), whereas a single case of CCRC, even in adults under 60 years of age, had a low positive predictive value (14.7%) in relation to P/LP variant detection. The variant c.208G>A;p.Glu70Lys was identified in four patients of East Asian ancestry only; the nonsense variant c.481C>T;p.Arg161* was found significantly more frequently in patients of Turkic ethnicities than in Slavic patients: 30.0 versus 2.5% (*p* = 0.0047). We did not identify significant differences in the profile of causative germline variants and frequencies of various VHLS clinical manifestations in our cohort compared with other Slavic people. Also, we examined 87 relatives of probands and identified 41 carriers of the causative variants. The obtained results may contribute to improving the diagnosis of VHLS.

## 1. Introduction

According to the International Agency for Research on Cancer, 20.6 million new cases of malignant tumors and 9.8 million deaths from them would be registered worldwide in 2024 [1]. About 10% of cancers are manifestations of hereditary tumor syndromes, diseases caused by germline mutations in oncogenes or tumor suppressor genes and characterized by an increased risk of tumors with certain types and localization. A number of syndromes among them develop not only malignant but also benign neoplasms, as well as non-neoplastic pathological changes in targeted organs. Moreover, germline causative (pathogenic and likely pathogenic) variants in genes involved in carcinogenesis can demonstrate different penetrance [2]. In this regard, development of the most effective methods for clinical examination of patients with suspected hereditary cancer syndromes, molecular genetic diagnostics, medical genetic counseling, surveillance and treatment is still relevant.

Von Hippel–Lindau syndrome (VHLS; OMIM #193300) is an autosomal dominant hereditary tumor syndrome named after the German ophthalmologist Johann von Hippel and the Swedish pathologist Arvid Lindau, who first described patients with the characteristic combinations of alterations in various organs and suggested that they were one disease in the early 20th century [3]. Common neoplasms of the syndrome include the retinal and central nervous system (CNS) hemangioblastomas, clear-cell renal carcinoma (CCRC), and pheochromocytoma. Depending on the presence of pheochromocytoma in the anamnesis, VHLS is divided into type 1 (without pheochromocytoma) and type 2 (with pheochromocytoma). In turn, type 2 is divided into subtypes depending on the tumor combinations: 2A (high risk of CCRC), 2B (low risk of CCRC), and 2C (pheochromocytoma only). Subtype 2C presents as a familial pheochromocytoma and requires differential diagnosis with other cancer syndromes, such as multiple endocrine neoplasia type 2 and SDH-deficiency cancer syndrome (hereditary paraganglioma/pheochromocytoma). Less frequently, VHLS is associated with pancreatic neuroendocrine tumors and other parts of the gastrointestinal tract, cystadenoma of the epididymis in men and the broad ligament of the uterus (mesosalpinx) in women, and tumors of the endolymphatic duct of the inner ear. Multiple cysts in the kidneys and/or pancreas are also relatively common manifestations of VHLS [4,5].

VHLS develops as a result of loss-of-function (LoF) heterozygous germline mutations in the *VHL* tumor suppressor gene localized in the 3p25 region. The second inactivating event during carcinogenesis is an extended deletion (loss of heterozygosity), aberrant methylation of the 5′-regulatory region, or a somatic point mutation in *VHL,* in accordance with the Knudson two-hit model. The protein (pVHL) is an important component of the multiprotein complex that degrades the heterodimeric hypoxia-inducible factor (HIF). In the absence of pVHL or the damage of its binding domains for HIF/elongin C (ELOC), the complex is not formed; HIF accumulates in the cell and activates the transcription of genes with HIF-responsive elements in the promoter, including those encoding growth factors and tyrosine kinase receptors that stimulate proliferation. Other possible mechanisms of the influence of *VHL* mutations on carcinogenesis have also been described, for example, inhibition of pRB, which facilitates the passage of the G1/S checkpoint, and a decrease in NF-kB signaling pathway activity [6,7]. It should be noted that some missense variants of *VHL* in the homozygous or compound heterozygous state, the most common of which is c. 598C>T;p.Arg200Trp, lead to hereditary erythrocytosis. This hereditary disease was originally described as Chuvash polycythemia in one of the ethnic regions of Russia (the Chuvash Republic). Missense variants of *VHL* in hereditary erythrocytosis patients were identified in other countries, for example, p.571C>G;p.His191Asp in Croatia. This disease is not associated with the development of solid tumors and presumably develops due to the distinct pVHL functions without dramatic accumulation of HIF, in particular, leading to stimulation of erythropoiesis by JAK2-STAT5 signaling [8].

The incidence of VHLS was previously estimated at 1:36,000 newborns, and still is in Orphanet [9] and some publications [2]. However, the current frequency of causative germline variants of VHL in Caucasians according to the gnomAD and ClinVar databases is 1:5000–9000, which is almost five times higher than assumed earlier [10]. Even considering the high but incomplete penetrance of the *VHL* mutations in different age groups (more than 90% of carriers of pathogenic germline variants manifest by the age of 60 [11]), the incidence of VHLS appears to have been significantly underestimated. The development of clinical diagnostic criteria for referring patients for *VHL* mutation analysis, optimization of molecular genetic diagnostics, analysis of phenotype–genotype associations in VHLS, and characteristics of pathogenic (probably pathogenic) germline variants of *VHL* remain relevant. According to our data, no comprehensive study of patients with VHLS has previously been performed in Russia.

We analyzed *VHL* gene mutations in 535 patients referred to the Research Center for Medical Genetics for VHLS diagnostics in the past ten years. The aim of our study was to investigate the phenotypic manifestations of VHLS, characterize germline *VHL* mutations, and identify genotype–phenotype associations. The study results made it possible to revise the role of CCRC as a clinical criterion for VHLS in adults under the age of 60, describe common causative missense variants of the *VHL* gene, and assess the proportion of various extended deletions within the *VHL* mutation spectrum in Russian patients.

## 2. Results

### 2.1. Characteristics of an Undifferentiated Cohort of Patients

**Figure 1:**
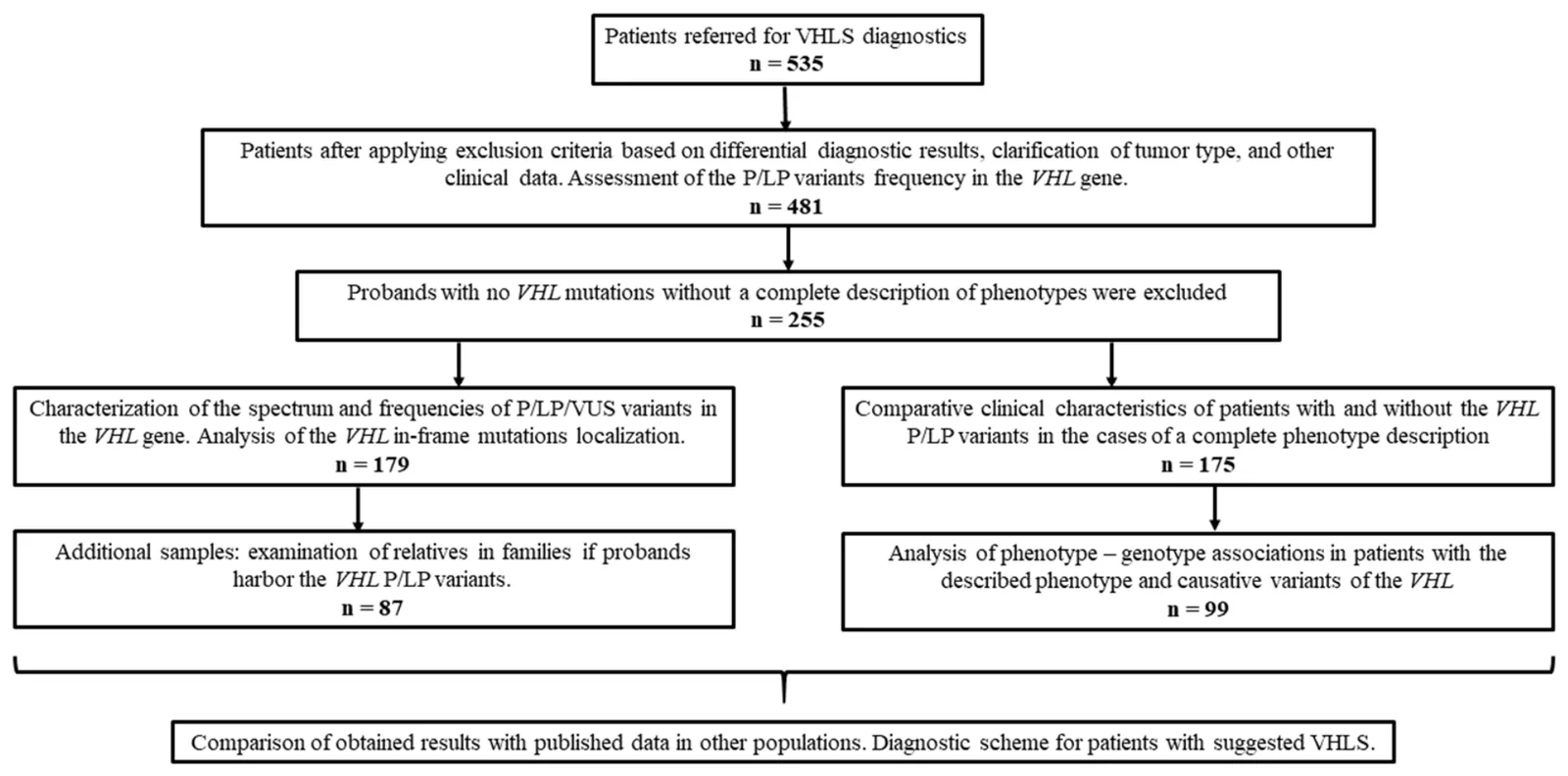
Study design. Legend. P/LP—pathogenic/likely pathogenic variant, VHLS—von Hippel–Lindau syndrome, and VUS—variant of uncertain significance.

Molecular genetic diagnostics of VHLS was performed for 535 primary patients. The incidence of causative *VHL* variants in the total cohort of patients was 33.1% (177/535). Exclusion criteria were applied based on the assessment of the compliance of referred cases with VHLS features, the results of differential diagnostics, and clarification of the tumor type (Figure 1).

Fifty-four patients were excluded. Among them, eight patients were over 60 years old, with a solitary tumor and without a family disease history; seven had retinitis, coloboma and other retinal pathologies not characteristic of VHLS; five had renal angiomyolipomas referred further for diagnosis of tuberous sclerosis; four presented with a negative result of validation in the blood of the mutation detected in the paraffin block with the tumor; four had an unspecified type of kidney or brain tumor; 14 patients had single neoplasms and cysts not associated with VHLS (angioma, glioblastoma, liver cysts, CNS or retroperitoneal cysts, bladder tumors, neck paraganglioma, parathyroid cancer, or multiple nevi); two patients had CCRC, chromophobe cancer and/or renal oncocytoma (confirmed cases of Birt–Hogg–Dubé syndrome with causative *FLCN* variants); and two cases had multiple endocrine neoplasia syndrome with bilateral pheochromocytoma and neuroendocrine tumors. There were single cases of patients with polycystic kidney disease and *PKD1* mutations; TFE3-associated renal-cell carcinoma; chromophobe renal-cell carcinoma; Fabry disease; mucopolysaccharidosis, with a pathogenic variant in *CHEK2*; a confirmed case of Li–Fraumeni syndrome with a pathogenic variant in *TP53;* and a case, previously described by us, of complex chromosomal pathology in a patient with Klinefelter syndrome and a balanced translocation involving chromosome 3 [12]. Causative variants in *VHL* were not identified in any of the excluded patients. The incidence of P/LP variants in *VHL* in the modified cohort was 36.8% (177/481).

Next, 217 patients with negative *VHL* testing results, whose samples were received from other clinics only for molecular genetic diagnosis of VHLS (or one of the diagnostic stages), without a description of phenotypes, were excluded from further analysis. A description of phenotypes, which allowed us to provide a comparative analysis, was available for 99 patients with the causative *VHL* variants and 76 patients without them, aged 11 to 73 years (Table 1). The age distribution of patients with the P/LP variants and without the identified *VHL* mutations did not differ significantly (Mann–Whitney test; *p* > 0.05). During the initial consultation, the proband was interviewed, and available medical records were analyzed to reveal a family history of cancer and identify the transmission of VHLS-associated diseases across generations. In total, 33 families with suspected hereditary forms of VHLS-associated diseases were identified.

### 2.2. Phenotypes of Patients with the Causative Variants and Non-Mutated VHL

**Figure 2:**
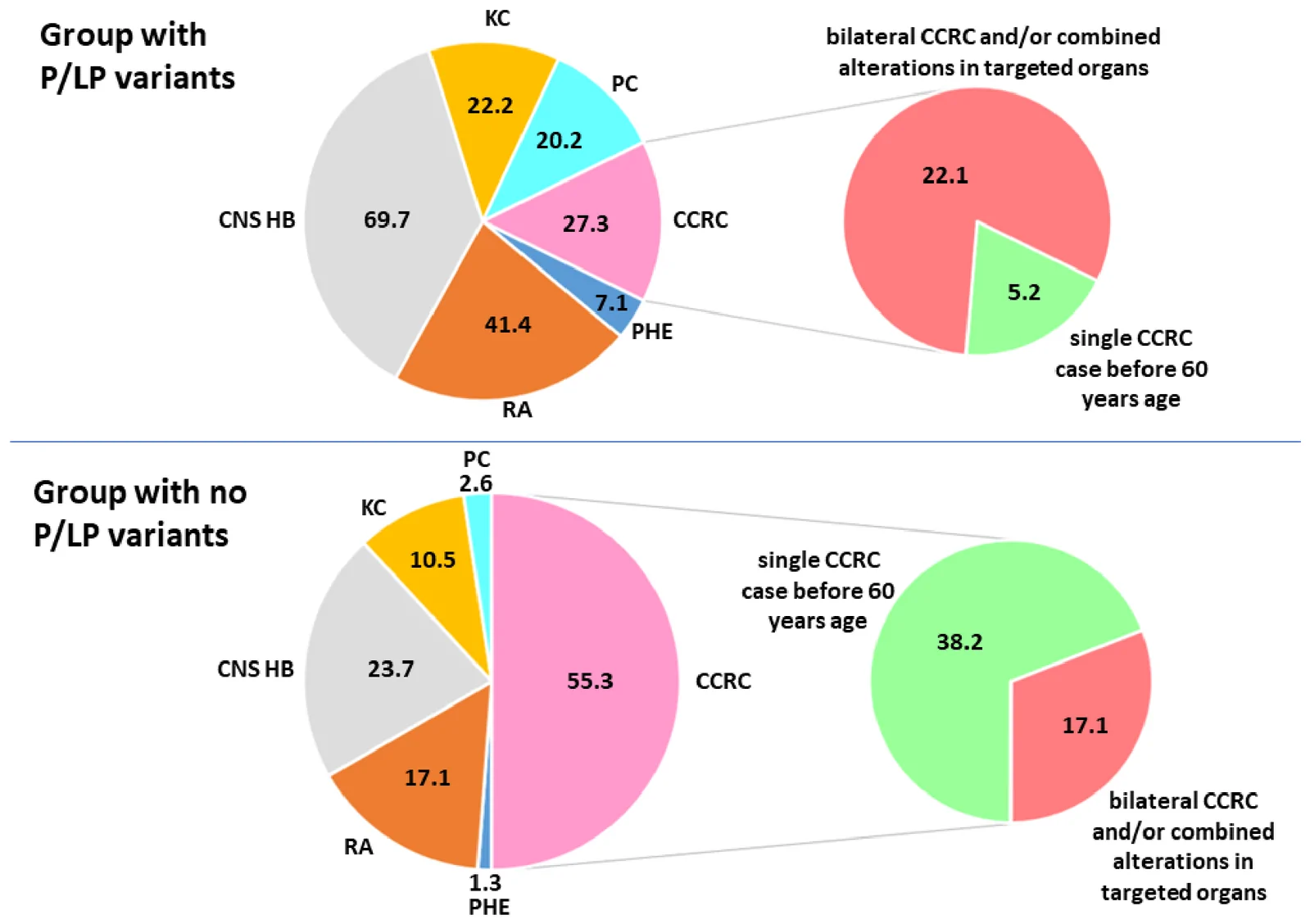
Comparison of clinical characteristics of patients with P/LP variants and without identified causative variants in the *VHL* gene. Legend: CNS hemangioblastomas are the most common manifestation of VHLS, whereas a CCRC without other signs of the syndrome is generally not associated with VHLS, even in adults under the age of 60; RA—retinal angiomatosis (hemangioblastomas), CNS HB—central nervous system hemangioblastoma, KC—kidney cyst, PC—pancreatic cyst, CCRC—clear-cell renal cancer, and PHE—pheochromocytoma; the relative frequency (%) of alterations in the compared groups is indicated.

Patients with causative variants of *VHL* and characterized phenotypes demonstrated the following VHLS clinical manifestations: CNS hemangioblastoma (69.7% (69/99)), retinal angiomatosis (hemangioblastoma) (41.4% (41/99)), CCRC (27.3% (27/99)), renal cysts (22.2% (22/99)), pancreatic cysts (20.2% (20/99)), and pheochromocytoma (7.1% (7/99)); pancreatic neuroendocrine tumors were detected in two patients and ovarian cystadenoma in one case. Liver hemangioma, liver cysts and retroperitoneal paraganglioma were comorbidities in single patients with VHLS. Combined pathological features (alterations in at least two targeted organs, multiple primary tumors, and/or a history of tumors in paired organs) were detected in 78.8% of patients with VHLS. The above-described distribution of the frequencies of pathological changes in patients with causative variants significantly differed from those in patients without identified P/LP variants of *VHL* (chi-square test, *p* < 0.01; Figure 2). Patients without P/LP variants demonstrated the following phenotypes: 55.3% (42/76) demonstrated CCRC (most of them were patients with a newly diagnosed solitary CCRC before the age of 60), 23.7% (18/76) CNS hemangioblastoma, 17.1% (13/76) retinal angiomas (hemangioblastomas), and 10.5% (8/76) renal cyst(s); pancreatic cyst(s) were observed in two cases, another two patients presented with a tumor of the endolymphatic duct of the inner ear, and there were single cases of patients with pheochromocytoma and neuroendocrine pancreatic tumors. A comparative analysis of the various VHLS manifestations showed that the most common tumors in patients with identified causative *VHL* variants were CNS and retinal hemangioblastomas. CCRC was detected in 27.3% of patients, of whom 25.9% (7/27) had bilateral tumors. CCRC was observed in most cases of the group without identified P/LP *VH*L variants; the percentage of cases with bilateral tumors was 11.9% (5/42). Bilateral alterations or a combination with other pathological changes in targeted organs in the group with CCRC were significantly more common in patients with the identified P/LP variant (two-tailed Fisher exact test, *p* = 0.00006), while isolated CCRC cases before the age of 60 in the anamnesis were predominantly in patients without genetically confirmed VHLS.

The frequency of primary multiple (combined) pathological changes in targeted organs was significantly higher in patients with causative variants than in the group without *VHL* mutations: 78.8% (78/99) versus 21.1% (16/76) (two-tailed Fisher's exact test; *p* < 0.0001). The positive predictive value of multiple lesions for a causative *VHL* variant was 83%. The relative risk was 3.2 (95% confidence interval (CI): 2.2–4.7), whereas for a solitary CCRC, the risks were 14.7% and 0.22 (95% CI: 0.09–0.5), respectively. The differences described above suggest that all patients with two or more clinical manifestations characteristic of VHLS should be referred for molecular genetic diagnostics, whereas patients with CCRC should be referred only in the presence of bilateral CCRC, a combination with other characteristic VHLS alterations, and/or a family history of the suggested VHLS; a single primary CCRC in an adult could be excluded from the clinical criteria for VHLS regardless of age.

### 2.3. Analysis of the VHL Germline Variants

**Figure 3:**
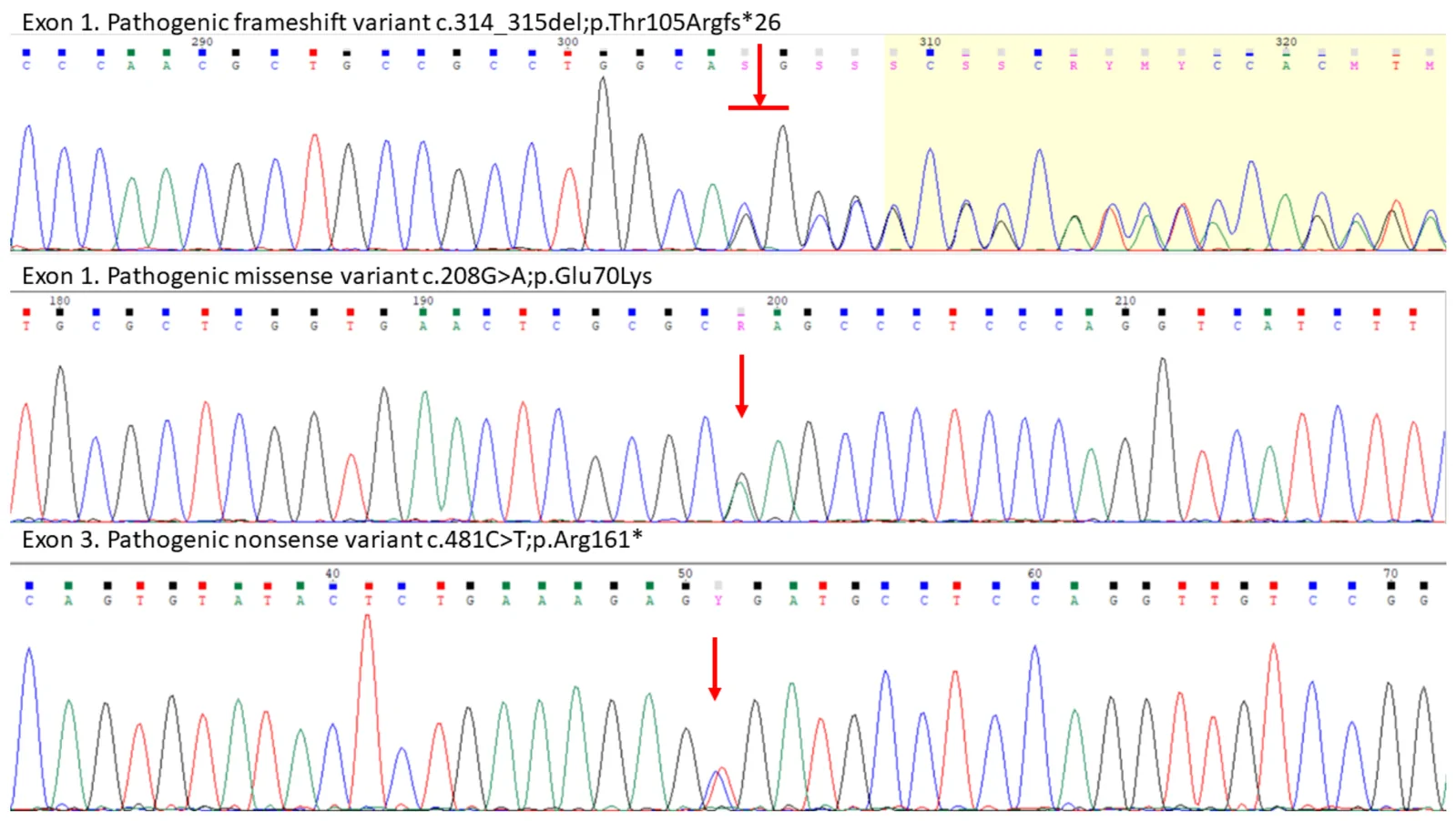
Sequencing of the pathogenic heterozygous variants in the *VHL* gene (indicated by arrows) belonging to different mutation types.

An analysis of point mutations in the coding region of the *VHL* gene was performed using Sanger sequencing in all patients referred for VHLS diagnosis at the first stage of the molecular genetic study (Figure 3). A total of 129 P/LP heterozygous variants and two variants of uncertain clinical significance (VUS) were identified (Table 2). Missense variants predominated, at 60.3% (79/131) among the identified variants, followed by nonsense variants at 18.3% (24/131), frameshift mutations at 8.4% (11/130), in-frame deletions/duplications at 6.9% (9/131), and splicing mutations at 6.1% (8/131). Variant pathogenicity was assessed using ACMG and CanVIG criteria. The CanVIG classification was preferred in cases of discrepancies, as it is more suited to the diagnosis of hereditary cancer syndromes and has specific guidelines for the *VHL* gene. From the three VUSs identified according to the ACMG classification, one was reclassified as an LP variant, and another was assigned the status of a “hot” VUS (one score to LP class), and functional analysis was recommended. Discrepancies in the scores between the common ACMG and CanVIG, excluding the VUS assessment, were observed in 11 cases. CanVIG generally allowed us to increase the pathogenicity class for rare frameshift and nonsense variants, taking into account the predicted nonsense-mediated mRNA decay (NMD).

The most frequently detected missense variants were in codon 167, at 12.4% (16/129) of causative variants; nonsense variant c.481C>T;p.Arg161* at 5.4% (7/129); and missense variants in codons 78 and 88 and phenylalanine deletion in codon 76 (c.227_229del;p.Phe76del) at 4.7% (6/129) in each case. Other germline P/LP variants were detected in fewer than four cases. Most often, causative variants were identified in the longest exon 1, at 42.6% (55/129) of cases; the frequencies of P/LP variants in other parts of the *VHL* gene were 35.7% (46/129) in exon 3, 15.5% (20/129) in exon 2, 4.7% (6/129) in the splice sites of intron 1 and 1.5% (2/129) of cases in the splice sites of intron 2. The mutation frequency of the *VHL* exons corresponded to their distribution by length (1 > 3 > 2).

In addition to patients with P/LP variants who were referred for VHLS diagnosis based on clinical criteria, the 5-year-old boy with the pathogenic variant c.98C>A;p.Ser33* was included. This variant was identified by exome sequencing due to diagnosis of Aarskog–Scott syndrome and reported as a secondary finding in accordance with ACMG recommendations [13] and was confirmed by Sanger sequencing. However, we lowered the initially assigned pathogenicity score according to ACMG based on CanVIG criteria for this variant and the substitution c.640T>A;p.*214Argext*14. The nonsense variant in the first case was localized before codon 54, so the PVS1 pathogenicity criterion could not be applied. The second variant was located outside the NMD region, and the functional significance of the pVHL sequence after codon 205 was unclear. The advantage of the CanVIG gene-specific guideline is detailed assessment of variants specifically in the *VHL* gene, which prevents overestimation of pathogenicity, as in the two cases discussed above, and, conversely, underestimation of pathogenicity for rare LoF variants. At the same time, the CanVIG recommendations are not integrated into widely used online pathogenicity calculators (Franklin, Varsome, and others) and require proper manual evaluation of each variant.

There was one case of mosaicism in the P/LP cohort with a mutant allele proportion in the blood of approximately 2.5% c.481C>T;p.Arg161*, which we described previously as a case study [14]. This patient developed hemangioblastomas of the retina, brain, and spinal cord from the age of 12 and also underwent removal of CCRC.

**Figure 4:**
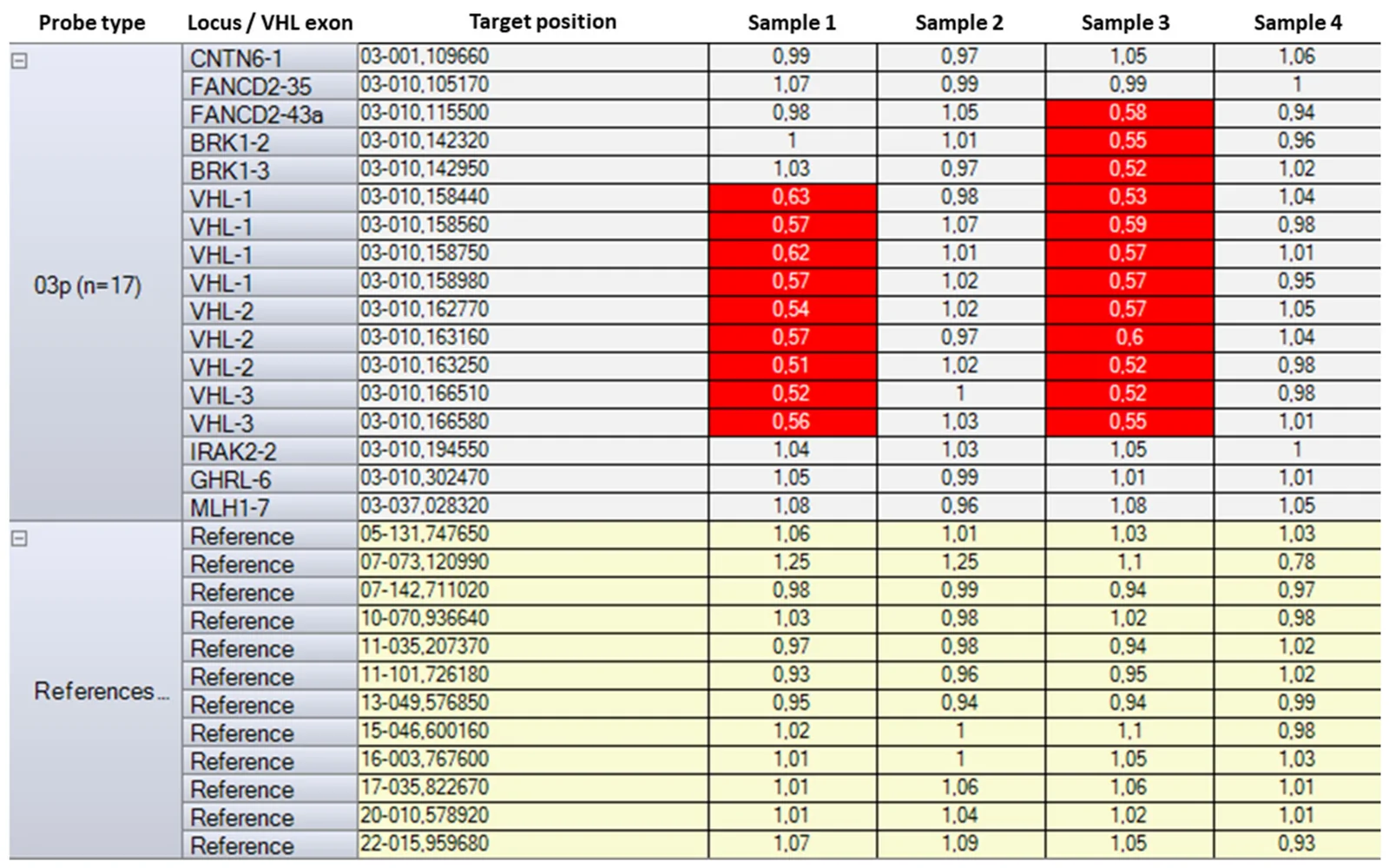
Detection of the *VHL* deletions by MLPA. Legend: A screenshot of the Coffalyser v.04 software is shown. The deletion of *VHL* exons 1–3 is detected in sample 1, while the deletion of *VHL* exons 1–3 and the 5′-flanking region is detected in sample 3; a 50% reduction in the signal from corresponding probes is marked by red automatically.

Patients without identified point variants in the *VHL* coding sequence were studied using MLPA (Figure 4).

**Figure 5:**
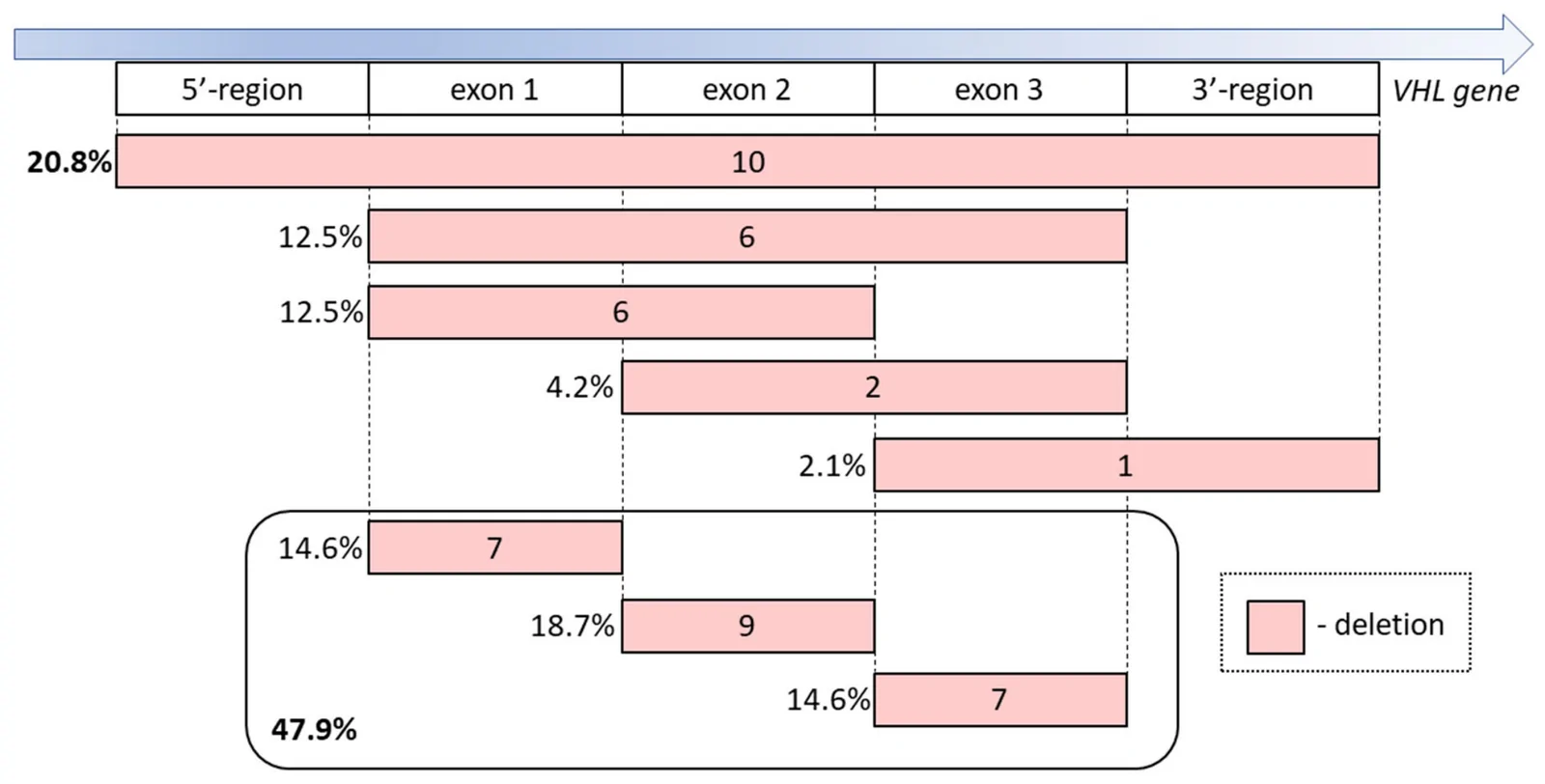
Extended *VHL* deletions detected in this study by MLPA. Legend: The most frequently detected type of deletion was that of the entire *VHL* gene and flanking regions on chromosome 3 (20.8% of cases), while various deletions of individual exons accounted for nearly half of all deletions identified by MLPA (47.9%); the number of cases is indicated within the deleted regions, with the relative frequency among all identified extended deletions shown alongside.

Extended heterozygous deletions were identified in 48 cases (Figure 5); no *VHL* duplications or other CNV types were detected. The frequency of extended deletions was 27.1% (48/177) of the *VHL* causative variants. Deletions of the entire gene with the promoter and flanking 5′- and 3′-sequences were detected in most cases (21%). Approximately 30% of deletions included two or more *VHL* exons (5′- or 3′-flanking sequence). It is noteworthy that deletions of single exons were detected with a similar frequency of 14–19%, accounting for a total of 47.9% of CNVs. This fact is significant for the choice of CNV detection method in favor of high-resolution MLPA, covering each exon with at least two pairs of probes, as it was done in our study. Furthermore, detection of heterozygotes for the deletion of only one probe in an exon requires further validation of this region using an independent method. Sometimes this situation is due to frequent neutral polymorphism at the probe hybridization site, which is a standard limitation of MLPA and is resolved by comparison with the sequencing data. However, it may also be due to the presence of a rare variant in a region that is not included in the sequenced part of *VHL*. We detected one case of such a heterozygous variant at the hybridization site of probe pair 02390-L16140. The ligation site was located 34 bp upstream of exon 2. Sanger sequencing of intron 1 adjacent to exon 2 revealed a heterozygous deletion c.341-44_341-18del, which we classified as VUS. Reclassification of this potential splicing variant by functional analysis is currently underway.

We categorized 177 probands with identified causative variants into groups of Slavic (159), non-Slavic European (4), Turkic (10), and East Asian (4) ancestry based on ethnicity data they reported about themselves. Comparison of mutation profiles in unrelated probands demonstrated two interesting features. First, the pathogenic missense variant c.208G>A;p.Glu70Lys was identified in four patients of East Asian ancestry only. Second, the nonsense variant c.481C>T;p.Arg161* was found significantly more frequently in patients of Turkic ethnicities than in Slavic patients: 30.0% (3/10) versus 2.5% (4/159) (*p* = 0.0047; two-tailed Fisher exact test). We did not identify other significant differences in the profile of causative germline variants compared with Caucasians as a whole.

**Figure 6:**
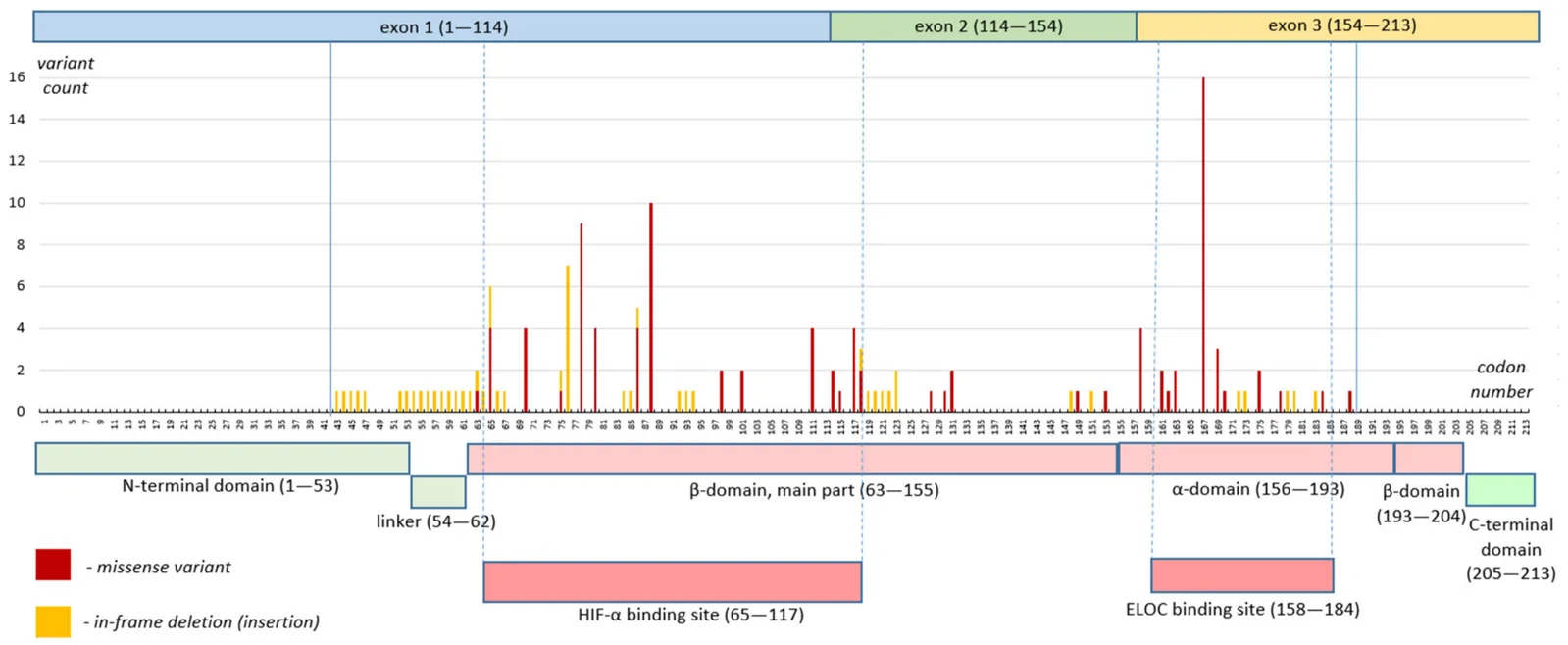
Localization of the in-frame P/LP variants in the *VHL* coding sequence. Legend: ELOC—elongin C; HIF—hypoxia-inducible factor. The exons encoding specific regions of pVHL are indicated at the top, while the pVHL domains are shown at the bottom. Domains affected by 0 to 2 in-frame *VHL* mutations are marked in green, whereas domains with 3 or more such variants are marked in red. The deep red color highlights the HIF-α- and ELOC-binding sites, which harbor 87.6% of in-frame mutations.

We placed a localization of in-frame germline variants (missense mutations and in-frame deletions/insertions (indels)) on the scheme of the *VHL* coding sequence. In addition to the P/LP variants identified in this study, the scheme contains germline in-frame P/LP and somatic (oncogenic) *VHL* variants in Russian patients with CCRC identified and published by us before the start of this study [15,16]. In total, the localization of 113 P/LP variants was analyzed, of which 83.2% (94/113) were missense mutations, and 16.8% (19/113) were in-frame indels (Figure 6). The variant’s position was associated with the *VHL* exons and functionally significant domains in the pVHL, the boundaries of which are designated in accordance with the schemes used by us and other authors previously [15,17]. Only two deletions were identified in the N-terminal domain, one of which also encompassed the β-domain. No in-frame variants were detected in the C-terminal region of pVHL and in the part of the β-domain encoded by exon 3. All causative missense variants and 95% (18/19) of indels were localized in the α- and β-domains of pVHL, which bind to ELOC and HIF-α, respectively. Moreover, the majority of the analyzed variants (87.6%) lead to changes directly in the binding sites for these pVHL partners: 54.9% (62/113) in the HIF-α-binding site and 32.7% (37/113) in the ELOC-binding site.

The distribution of 99 patients with known clinical characteristics and causative *VHL* variants with regard to the VHLS type and variant type was analyzed. Variant types were either LoF (nonsense, frameshift, splicing variants, and deletions detected by MLPA) or gain-of-function (GoF) variants (missense and in-frame indels). Due to the small number of patients with pheochromocytoma, we did not divide type 2 VHLS into subtypes. Although the relative frequency of GoF variants was higher in patients with type 2 VHLS compared with type 1, the differences were not significant: 48.9% (45/92) versus 85.7% (6/7). This may be due to the small sample size of patients with pheochromocytoma, which may be explained by the specialization of the clinics referring patients for VHLS diagnostics to our center.

### 2.4. Examination of the Probands’ Relatives

**Figure 7:**
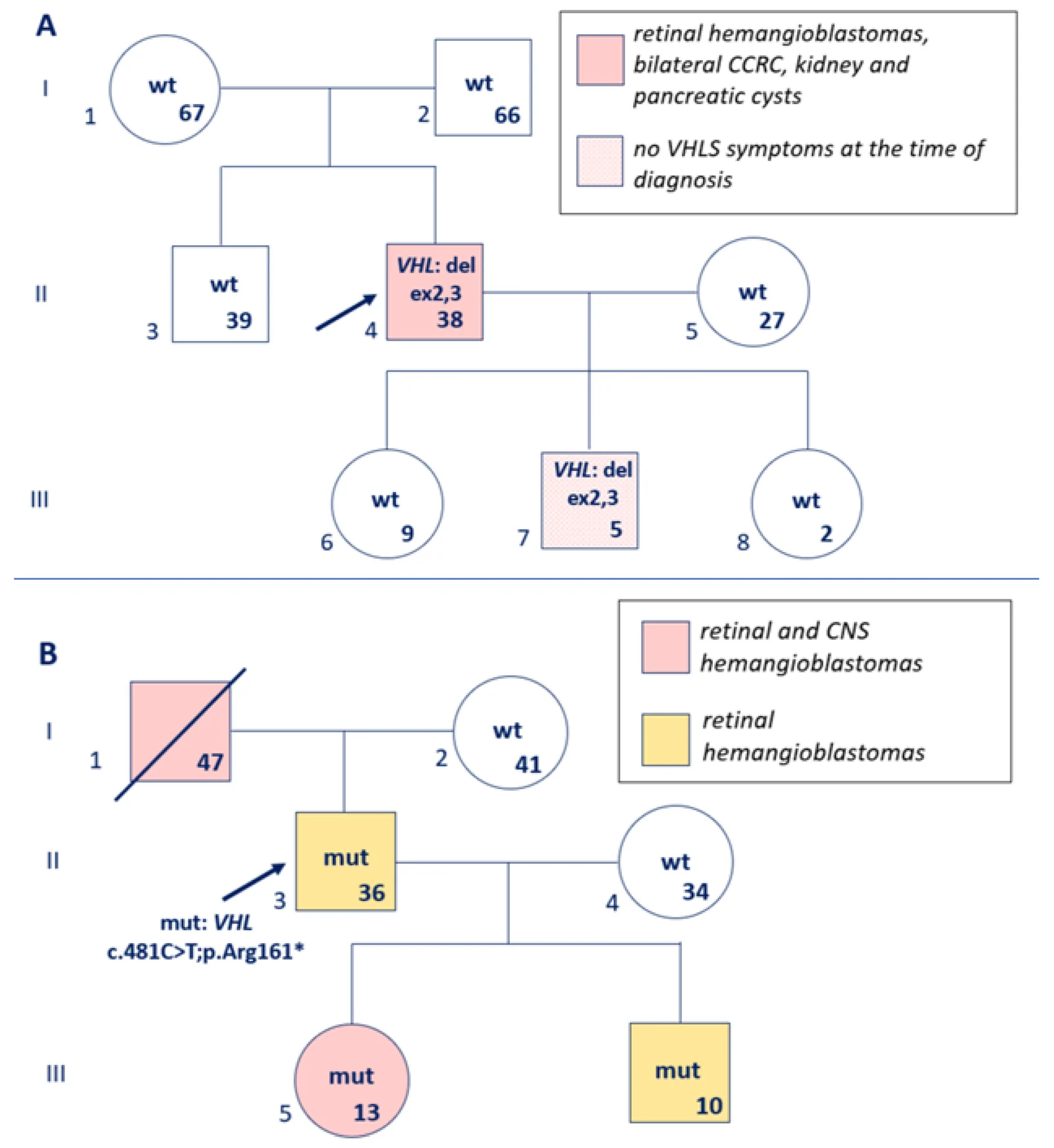
Pedigrees of patients with VHLS. Legend: (**A**)—a case of de novo deletion of *VHL* exons 2 and 3: the 38-year-old proband with bilateral CCRC and *VHL* exons 2–3 deletion; his parents had no VHLS symptoms and this deletion, but exons 2–3 deletion was inherited by his 5-year-old asymptomatic son. (**B**)—VHLS type 1 with the frequent development of retinal angiomatosis (hemangioblastomas): all affected family members from 10 to 47 years old with the mutation had retinal hemangioblastomas. mut—the *VHL* P/LP variant; wt—“wild type”—normal genotype without causative *VHL* variants. Probands are indicated by arrows.

After molecular genetic diagnostics in probands, we examined their 87 relatives for the identified P/LP *VHL* variants. Causative variants were detected in 25 close relatives: asymptomatic mutation carriers, including young adults (brothers and sisters), and, as a rule, children of primary patients (Figure 7). The proportion of inherited cases in the patients’ children was 46.7% (21/45) and did not differ significantly from the theoretically expected frequency of VHLS in the first generation. Moreover, 16 relatives of probands with VHLS symptoms who had not previously been diagnosed with the disease (siblings, parents, and second-degree and more distant relatives) were identified. We also examined families in which the proband presented with non-neoplastic manifestations of VHLS, for example, the family of a 29-year-old woman with polycystic kidney and pancreatic disease, previously described by us as a case study. We identified the pathogenic *VHL* variant c.203C>A;(p.Ser68*), which was subsequently detected in the patient’s 34-year-old sister with pancreatic cysts and in the patient’s 6-year-old daughter without VHLS symptoms (the patient’s father died before examination; he had CNS hemangioblastomas and CCRC) [18]. Overall, P/LP variants were detected in 47.1% (41/87) of relatives of primary patients. The obtained results allowed us to begin timely monitoring of individuals with causative *VHL* variants in families.

## 3. Discussion

### 3.1. Manifestation and Clinical Heterogeneity of VHLS

Von Hippel–Lindau syndrome is characterized by clinical heterogeneity. We observed the following frequencies of VHLS manifestations in the cohort of primary patients: CNS hemangioblastoma at 70%, retinal angiomatosis (hemangioblastomas) at 41%, CCRC at 27%, renal cysts at 22%, pancreatic cysts at 20%, pheochromocytoma at 7%, pancreatic neuroendocrine tumors at 2%, and ovarian cystadenoma at 1%. The distribution of clinical manifestations of VHLS in patients with the characterized phenotypes in our study did not generally differ significantly from the frequencies of pathological changes reported by other authors [19,20]. The lifetime relative risk of developing pathological changes in patients with VHLS reported by a recent systematic review was higher: CNS hemangioblastoma at 60–80%, retinal hemangioblastoma at 25–60%, CCRC at 17–75%, renal cysts at up to 42%, pancreatic cysts at up to 85%, pancreatic neuroendocrine tumors at 10–17%, and pheochromocytoma at 10–25% [5]. For example, the frequency of pheochromocytoma was 7.1% in our cohort, although, according to a number of studies, its lifetime occurrence in VHLS is approximately 10–30% [7,21]. These differences can be explained by two considerations. First, the percentage of children and young adults under 25 years of age among our patients with P/LP variants and characterized phenotypes was 25.3% (25/99), and these patients remain at risk for developing pheochromocytoma later. Second, patients were referred to our center for VHLS diagnostics from clinics specializing in the diagnosis and treatment of CNS tumors, ophthalmic oncology, and urological oncology. It is essential to consider correlations between the renal tumor type and the candidate gene to search for the germline variant. This often saves time and labor costs during diagnostics, especially in cases of identified molecular subtypes of renal-cell carcinoma [22].

This study has several limitations. It was a retrospective study conducted at a single genetic center without external validation. The patients included in this study were primarily referred by clinics specializing in CNS tumors and ophthalmological and urological diseases; consequently, our cohort may have lacked patients with rare manifestations of VHLS, such as pancreatic or inner-ear tumors and pheochromocytoma. For example, the proportion of pancreatic neuroendocrine tumors in other studies reached 5–12% [23], enabling the authors to identify an association between the risk of these tumors and point mutations in exon 3 of the *VHL* gene; notably, a significant portion of their patients were from Eastern European countries [24]. Furthermore, we lacked additional patient information (preliminary VHLS diagnosis only) about patients with negative results of molecular genetic testing if other examinations had been performed outside our institution. Only 68.6% (175/255) of patients were confirmed VHLS cases and/or underwent the necessary VHLS-targeted organ evaluations and were included in the analysis of genotype–phenotype associations due to the completeness of clinical data.

### 3.2. Spectrum and Frequency of Germline VHL Variants in Different Countries and Types of VHLS

Missense variants covered more than 50% of causative variants in the mutation distribution in our patients, which is consistent with data from other authors diagnosing VHLS in a targeted cohort [19]. It should be noted that somatic *VHL* mutations are the most common oncogenic alteration in sporadic CCRC, but they are represented in the vast majority by LoF indel variants, while missense variants are rare [15]. So, the spectrum of somatic *VHL* mutations in CCRC is closer to germline variants in VHLS type 1 than type 2. A study of patients with VHLS and a high proportion of CCRC by other authors demonstrated a higher frequency of germline LoF variants, rather than missense GoF variants [25].

De novo germline *VHL* mutations may account for up to 20% of VHLS cases [10]. In our study, only one case of a de novo variant, a deletion encompassing the *VHL* exons 2–3 (Figure 7A), was obtained. However, it should be noted that after identifying P/LP variants in 177 probands, parental testing was generally unnecessary. Regarding two cases with VUS, such testing could not be performed due to the unavailability of material from both parents. Therefore, the true frequency of de novo cases in our cohort could not be estimated.

More than 1000 pathogenic (oncogenic) variants in the *VHL* gene have been described. VHLS type 1 has been shown to be associated with LoF variants (nonsense, splicing, and frameshift mutations), while type 2 is associated with missense variants [26]. No differences were found between VHLS types depending on the functional classification of causative variants, possibly due to the small number of patients with VHLS type 2 in our study. However, a meta-analysis of VHLS in Europe, Asia, and America revealed a high frequency of LoF variants with a premature stop codon in VHLS type 1 versus 2. The frequency of causative missense variants also differed: 58.9% in VHLS type 1 versus 88.1% in type 2 [27]. Interestingly, the concentration of HIF in cells with mutated *VHL* and different VHLS types decreases in the following order: type 1 > type 2B > type 2A > type 2C [6]. Localization of the frequent P/LP missense variants is similar in patients with VHLS in different ethnic groups. Thus, a study of 577 patients from 211 families with VHLS in China demonstrated that the first 10 codons with the causative missense *VHL* variants in unrelated patients, in descending order of frequency, are 167 > 65 > 86 > 78, 88, 117, 162 > 80, 90, 161, and then variants in solitary cases [17]. The first 10 codons with P/LP missense variants in our patients coincided in frequency with the work cited above by 80%: 167 > 88 > 78 > 65, 70, 80, 86, 111, 117, and 158. Moreover, patients were predominantly of East Asian origin in the cited study, while they were of Slavic Caucasian origin in our work, and only in some cases were there families of Asian origin (for example, one Korean patient with the missense variant c.208G>A;p.Glu70Lys, which is characterized by the founder effect and is the most frequent pathogenic missense variant in the Korean population [28]). According to a meta-analysis of patients from different populations, substitutions in codon 167 are the most frequent, followed by codons 65 and 98 [27]. A recent analysis of VHLS from 40 centers characterized 432 causative germline variants, with the most frequent missense variants located at codons 78, 98, 161, and 167. The authors demonstrated that the c.292T>C;p.Tyr98His mutation has a founder effect in southern Germany and is associated with a lower risk of multiple VHLS symptoms than other P/LP *VHL* variants. The same study also demonstrated that the p.Arg161* variant is associated with an increased risk of CCRC [29]. These results, together with our work, once again emphasize the significance of in-frame variants in the HIF-α- and ELOC-binding sites and the advisability to reclassify all VUS located in these sites for VHLS diagnostics.

We did not observe missense variants of *VHL*, which were previously identified as causes of hereditary erythrocytosis (Chuvash polycythemia), in our cohort. Currently, homozygotes or compound heterozygotes for the variants p.Arg200Trp, pHis191Asp, p.Leu188Val, p.Pro192Ala, p.Asp126Asn, p.Pro138Leu, p.Gly144Arg, and p.Asp143= have been described in hereditary erythrocytosis. Interestingly, the last mutation leads to skipping of exon 2, but has a weaker effect on splicing [8] than, for example, the variant in the same exon c.414A>G;p.Pro138=, which we identified in one patient with VHLS. Characterization of the *VHL* germline variants in different countries remains a relevant task. It allows us to identify the most common point mutations in different ethnic groups (regions).

We compared *VHL* mutations in our study with the data from other authors who investigated patients with VHLS in Slavic patients. It should be noted that the proportion of CNVs among the causative variants in our study (27.1%) differed from Bulgarian (20%) or Polish (40%) cohorts; however, this discrepancy may be explained by the use of different methods for detecting extended deletions [30,31]. Interestingly, the deletions extended into the 5′-flanking region of the *VHL* gene (Figure 4 and Figure 5) in some of our patients and encompassed the locus with the *FANCD2* tumor suppressor gene, which is involved in DNA repair. A Polish case study previously reported a woman with VHLS and bilateral breast cancer diagnosed before age 50 who carried a similar extended deletion; the authors hypothesized that the *FANCD2* disruption might be associated with an increased risk of malignancy [32]. However, whole-genome analysis is needed to clarify this hypothesis and exclude other candidate genes. Overall, the majority of reported VHLS cases among Slavic patients since the early 2000s were published by colleagues from Poland. As more data accumulate, it will be possible to provide a comparative analysis of mutation frequencies across Eastern European Slavic countries.

### 3.3. Rare Genetic Aberrations Causing VHLS and Requiring Advanced Molecular Genetic Testing

Causative germline variants in the *VHL* gene are detected in an average of 95% of patients with a combination of characteristic VHLS features. However, there remain patients without germline P/LP variants in *VHL*. These patients may have mosaic forms of the disease and a low blood variant allele frequency, too small for detection by Sanger sequencing, below the variant allele cut-off level of genome, exome, or gene panel sequencing for germline variants. Mutations in other minor candidate genes whose products directly interact with pVHL for HIF degradation, such as *ELOC* and *HIF2A*, cannot be ruled out [33]. Finally, the presence of pathogenic intronic variants and changes in the regulatory noncoding regions of VHL cannot be ruled out. These are also variants not included in the analyzed regions or are classified as VUS due to insufficient data about their pathogenicity.

Our study included a patient with a pathogenic mosaic variant of *VHL* who developed multiple retinal and CNS hemangioblastomas and CCRC [14]. Cases of mosaicism in patients with VHLS have been previously published by other authors [34,35]. According to some estimates, the proportion of mosaic *VHL* mutations in VHLS may reach up to 4–5% [36]. However, the incidence of mosaicism is difficult to estimate because some cases may mimic sporadic neoplasms. Material from two or more different primary tumors is not always available to search for a mosaic variant and validate it in blood or other normal tissue. Performing targeted deep high-throughput sequencing in every case referred to a laboratory for *VHL* mutation testing after a negative routine result is currently too expensive. However, it is possible to test cases suspected of mosaicism in specialized laboratories that will perform deep sequencing of the entire *VHL* coding sequence, although even in this case, there remains the possibility of missing rare *VHL* mutations in non-coding regions. Whole-genome sequencing of patients with negative routine *VHL* testing appears justified, allowing for the identification of causative variants in non-coding regions and differential diagnosis with other hereditary cancer syndromes. In particular, a balanced translocation t(1;3)(p36.3;p25) with a breakpoint in the *VHL* second intron was identified in a patient with CCRC, cerebellar hemangioblastoma, and polycystic kidney and pancreatic disease [37].

The *ELOC* gene encoding elongin C (a key pVHL partner in the construction of the multiprotein complex) is considered a second candidate gene for VHLS, in addition to *VHL*. To date, patients with VHLS caused by *ELOC* germline missense variants have been reported. The severity and combined alterations of VHLS in those patients are no different from those in VHLS type 1 with the *VHL* LoF variants. The amino acid substitutions are located in the ELOC-binding site with pVHL, and these variants, as somatic mutations, determine the new molecular subtype of kidney cancer: ELOC-associated renal-cell carcinoma in correspondence with the World Health Organization classification since 2022 [38,39].

Surveillance and treatment options in patients with VHLS are discussed in the Supplementary Material.

## 4. Materials and Methods

**Samples.** This study included genomic DNA samples from 535 primary patients referred for VHLS diagnostics from December 2015 to May 2026 at the Research Center for Medical Genetics, comprising 290 women and 245 men aged 1 to 79 years; the mean age of patients (standard deviation) was 36.2 ± 12.4 years. The inclusion criteria for this study were one or more diseases in the anamnesis: CNS hemangioblastoma, retinal angiomatosis (hemangioblastomas), CCRC, renal and/or pancreatic cysts, pheochromocytoma, pancreatic neuroendocrine tumor, an endolymphatic sac tumor of the inner ear, and a preliminary clinical diagnosis of VHLS in other clinics without specifications (Figure 1). The main medical centers that referred patients for VHLS diagnostics included the Blokhin National Medical Research Center of Oncology, the National Medical Research Center of Radiology, the Helmholtz National Medical Research Center for Ophthalmic Diseases, the Medical Genetic Center “Genomed”, and the National Medical Research Center of Neurosurgery named after academician N.N. Burdenko. Subsequently, patients without a pathologically confirmed tumor type, patients over 60 years of age with only one of the possible VHLS features without a family history of the disease, and patients with a clarified diagnosis that differed from the initial one (e.g., chorioretinitis instead of angiomatosis, or renal angiomyolipomas instead of CCRC) were excluded from our cohort. The sample size after applying the clinical exclusion criteria was 481 samples, from which patients without *VHL* mutations or with incomplete phenotype descriptions were then removed for further analysis. The final sample size, which was used in a comparative analysis across various parameters, was 255 primary patients. After VHLS diagnosis was confirmed, we additionally analyzed the identified P/LP variants in 87 relatives of the probands.

**DNA isolation.** Blood was collected into tubes containing EDTA. Genomic DNA was then isolated from the blood using the ExtractDNA Blood & Cells kit (“Eurogen”, Moscow, Russia), according to the manufacturer’s instructions. The concentration of the extracted DNA was determined fluorimetrically using a Qubit 3.0 instrument and the Qubit™ dsDNA HS (High Sensitivity) Assay Kit (Thermo Fisher Scientific, Waltham, MA, USA).

**PCR and Sanger sequencing.** Exons 1–3 of the *VHL* gene were amplified by polymerase chain reaction (PCR). The reaction mixture consisted of 50–100 ng of genomic DNA, 2.5 mM of MgCl_2_, 1.5 mM of each dNTP, 2 pmol of forward and reverse primers, 1 unit of thermostable Taq-polymerase, and 5 μL of 10x PCR buffer (SibEnzyme, Novosibirsk, Russia); the volume of the mixture was 25 μL. PCR was performed in a C1000 Touch Thermal Cycler (Bio-Rad Laboratories, Hercules, CA, USA). The primer sequences have been published previously [15]. PCR products were treated with 2 u.a. of E. coli exonuclease I (Fermentas, Vilnius, Lithuania) and 1 u.a. of alkaline phosphatase from calf intestine (SibEnzyme, Novosibirsk, Russia), involving incubation for 1 h at 37 °C, followed by enzyme inactivation at 85 °C for 15 min. Sanger sequencing was performed using the BigDye^®^ Terminator v3.1 Cycle Sequencing Kit according to the manufacturer’s instructions (Applied Biosystems, Foster City, CA, USA). Fluorescent products were detected with a 3500 Genetic Analyzer (Thermo Fisher Scientific, Waltham, MA, USA). Sequencing chromatograms were analyzed using the Chromas v.2.6.6 software (Technelysium, Brisbane, Australia).

**MLPA for detection of CNVs in the *VHL* gene.** Multiplex ligation-dependent probe amplification (MLPA) was used to detect CNVs in *VHL* spanning one or more exons. The SALSA MLPA Probemix P016 probe kit and other reagents required for hybridization, ligation, and amplification were obtained from MRC Holland (Amsterdam, The Netherlands). This kit contained 17 probes in the region of *VHL* localization (9 encompassed exons, 6 were in the flanking regions and 2 were centromeric and telomeric on 3p, respectively), as well as 12 reference probes for other regions of the genome not involved in CNVs in patients with VHLS. MLPA was performed according to the manufacturer’s protocols. The MLPA reaction contained 50–200 ng of genomic DNA. Fragment analysis was performed with a 3500 capillary genetic analyzer (Thermo Fisher Scientific, Waltham, MA, USA). The peak intensity and distribution in the chromatogram were analyzed using the Coffalyser v.04 software (MRC Holland, Amsterdam, The Netherlands); a heterozygous deletion corresponded to a value of 0.40 < FR < 0.65.

**Pathogenicity of genetic variants.** Genetic variants were designated according to the HGVS (Human Genome Variation Society) nomenclature [40]. The pathogenicity of germline variants was determined according to the ACMG (American College of Medical Genetics and Genomics) criteria [41,42] using the Franklin (https://franklin.genoox.com/clinical-db/home (accessed on 26 August 2026)), ClinVar (https://www.ncbi.nlm.nih.gov/clinvar/ (accessed on 26 August 2026)), and Varsome (https://varsome.com/ (accessed on 26 August 2026)) databases, and the pathogenicity calculator of the Research Center for Medical Genetics (http://calc.generesearch.ru/ (accessed on 26 August 2026)). The UCSC (https://genome.ucsc.edu/ (accessed on 26 August 2026)) and Ensembl (https://www.ensembl.org/ (accessed on 26 August 2026)) genome browsers were used to visualize genetic variants. The AlpaMissense (https://alphamissense.hegelab.org/ (accessed on 26 August 2026)) and PolyPhen2 (https://genetics.bwh.harvard.edu/pph2/bgi.shtml (accessed on 26 August 2026)) databases were used to predict in silico the functional significance of missense variants, while the effect of variants on splicing was predicted using the programs SpliceAI (https://spliceailookup.broadinstitute.org/ (accessed on 26 August 2026)) and MaxEntScan (https://genebe.net/tools/maxentscan (accessed on 26 August 2026)). Causative variants included pathogenic (P) and likely pathogenic (LP) genetic variants. Considering the more detailed pathogenicity assessment of germline variants in the gene-specific guidelines of CanVIG (Cancer Variant Interpretation Group, UK) for *VHL*, we also annotated variants according to these specifications [43]. The pathogenicity of CNV was determined according to the ACMG/ClinGen recommendations [44].

**Statistical analysis.** The absolute frequencies of clinical features in groups were compared using the nonparametric Mann–Whitney test, χ2, and the two-tailed Fisher’s exact test (the number of patients with different VHLS manifestations in groups with and without causative *VHL* variants; α = 0.05) using Excel (Microsoft Corporation, Redmond, WA, USA) and STATISTICA v.10 (StatSoft, Tulsa, OK, USA).

## 5. Conclusions

**Figure 8:**
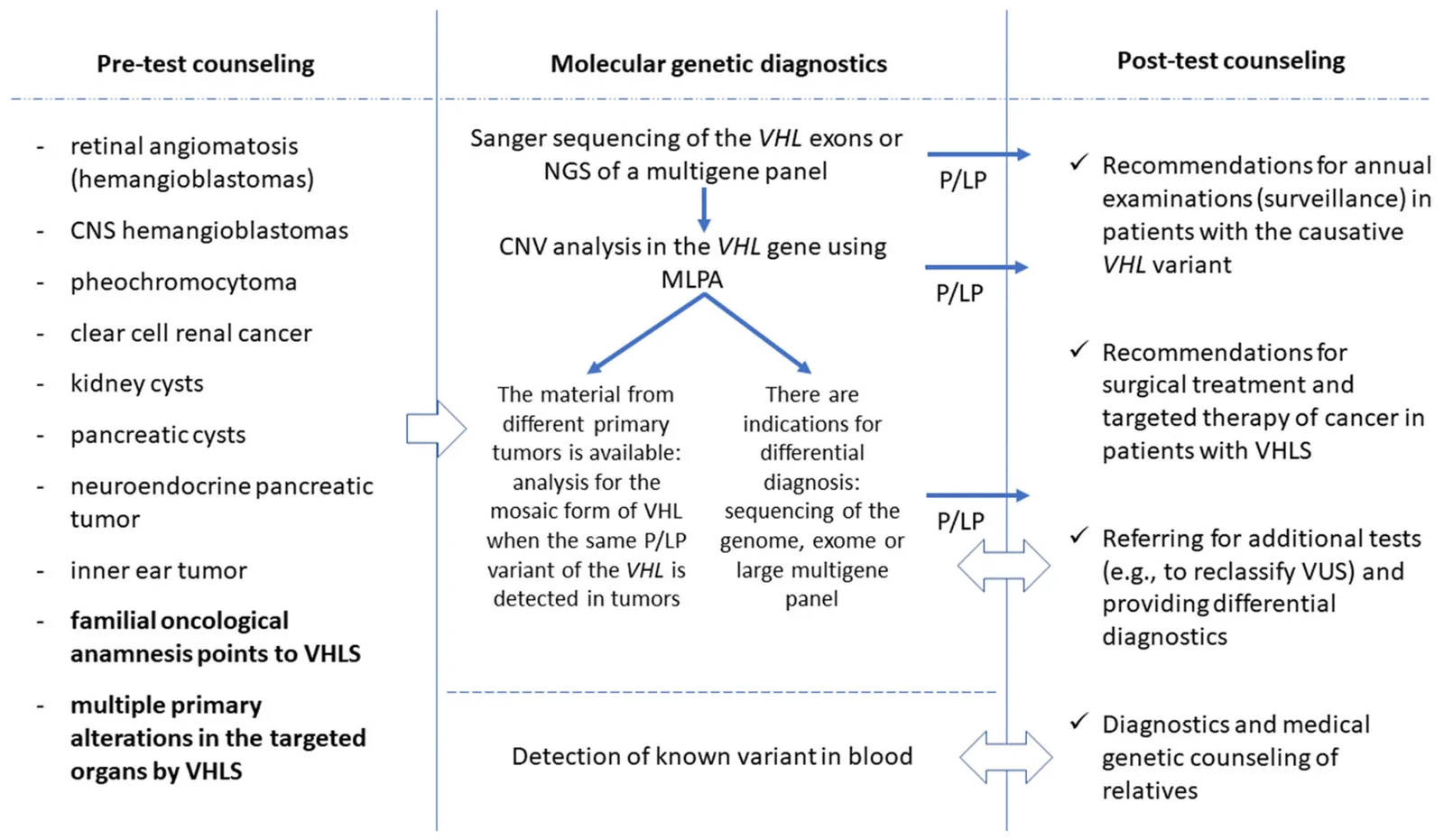
Scheme for genetic diagnostics of VHLS. Legend: NGS—next-generation sequencing, CNS—central nervous system, CNV—copy number variation, MLPA—multiplex ligation-dependent probe amplification, P/LP—pathogenic/likely pathogenic genetic variant, VHLS—von Hippel–Lindau syndrome, and VUS—variant of uncertain significance.

Thus, pre-test genetic counseling remains essential for molecular genetic diagnosis of VHLS, with assessment of the complex involvement of target organs. Two or more CNS hemangioblastomas are a significant indication for molecular genetic diagnosis, whereas a solitary newly diagnosed CNS hemangioblastoma without other signs of the disease is unlikely to be related to VHLS. Mutation testing in a patient suspected of having VHLS should include, at a minimum, sequencing of the coding region and searching for CNVs in the *VHL* gene, optionally supplemented by mosaicism analysis in the presence of the same *VHL* mutation in different primary tumors, or genome sequencing in the case of a negative result at the first stage of VHLS diagnostics (Figure 8). Post-test consultation with a geneticist is necessary for the possible differential diagnosis of VHLS with other hereditary tumor syndromes, additional tests for the reclassification of VUS, testing relatives who are possible carriers of the causative variant, recommendations about surveillance for carriers of P/LP variants, and recommendations on the specifics of surgical treatment and targeted therapy for cancer in patients with VHLS.

## Figures and Tables

**Table 1:** Patients’ characteristics (*n* = 255).

Parameter	Name	Number of Cases	%
Sex	Men	119	46.7
	Women	136	53.3
VHLS-associated disease	CNS hemangioblastomas	87	34.1
	Retinal angiomatosis	54	21.2
	Clear-cell renal cancer	68	26.7
	Pheochromocytoma	8	3.1
	Neuroendocrine pancreatic tumor	3	1.2
	Inner air (endolymphatic sac) tumor	2	0.8
	Broad ligament cystadenoma	1	0.4
	Renal cysts	30	11.8
	Pancreatic cysts	22	8.6
	Multiple alterations	94	36.7
	Suspected VHLS, no features provided	80	31.4
Family history	VHLS-associated disease	33	12.9

Legend: VHLS—von Hippel–Lindau syndrome.

**Table 2:** P/LP point variants in the *VHL* gene identified in this study by sequencing.

*VHL* Exon/Intron	Germline Variant (RefSeq NM_000551.4)	Number of Cases	Pathogenicity ACMG/CanVIG	Mutation Type
Exon 1	c.98C>A;p.Ser33*	1	P/LP	Nonsense
	c.123_137del;p.Ser43_Glu47del	1	VUS/LP	IDI
	c.175_196del;p.Pro59*	1	LP/P	Nonsense
	c.194C>T;p.Ser65Leu	2	P/P	Missense
	c.188T>G;p.Leu63Arg	1	LP/LP	Missense
	c.203C>A;p.Ser68*	1	P/P	Nonsense
	c.204dup;p.Arg69alafs*63	2	P/P	Frameshift
	c.208G>A;p.Glu70Lys	4	P/P	Missense
	c.213_215delinsTT;p.Q73Rfs*54	1	LP/P	Frameshift
	c.219G>C;p.Gln73His	1	VUS/hot VUS	Missense
	c.223_228del;p.Ile75_Phe76del	1	LP/P	IDI
	c.224T>G;p.Ile75Ser	1	LP/LP	Missense
	c.227_229del;p.Phe76del	6	P/P	IDI
	c.227_228delinsAA;p.Phe76*	1	LP/P	Nonsense
	c.233A>G;p.Asn78Ser	4	P/P	Missense
	c.233A>T;p.Asn78Ile	2	P/P	Missense
	c.238A>C;p.Ser80Arg	1	P/P	Missense
	c.239G>T;p.Ser80Ile	1	P/P	Missense
	c.239G>A;p.Ser80Asn	2	P/P	Missense
	c.256C>T;p.Pro86Ser	2	P/P	Missense
	c.256C>G;p.Pro86Ala	1	P/P	Missense
	c.257C>T;p.Pro86Leu	1	P/P	Missense
	c.262T>A;p.Trp88Arg	4	P/P	Missense
	c.262T>G;p.Trp88Gly	1	P/P	Missense
	c.264G>C;p.Trp88Cys	1	P/P	Missense
	c.264G>A;p.Trp88*	1	P/P	Nonsense
	c.280G>T;p.Glu94*	1	P/P	Nonsense
	c.294C>G;p.Tyr98*	1	P/P	Nonsense
	c.294C>A;p.Tyr98*	1	P/P	Nonsense
	c.293A>G;p.Tyr98Cys	1	P/P	Missense
	c.306_312del;p.Pro103Argfs*54	1	LP/P	Frameshift
	c.314_315del;p.Thr105Argfs*26	1	P/P	Frameshift
	c.331A>G;p.Ser111Gly	2	P/P	Missense
	c.331A>T;p.Ser111Cys	1	P/P	Missense
	c.340G>T;p.Gly114Cys	1	LP/P	Missense
Intron 1	c.340+1G>T	1	LP/P	Splicing
	c.340+1G>C	1	P/P	Splicing
	c.340+1G>A	2	P/P	Splicing
	c.341-2A>C	1	P/P	Splicing
	c.341-22_343del	1	LP/P	Splicing
Exon 2	c.344A>G;p.His115Arg	1	P/P	Missense
	c.350G>C;p.Trp117Ser	2	P/P	Missense
	c.351G>T;p.Trp117Cys	2	P/P	Missense
	c.353T>C;p.Leu118Pro	1	P/P	Missense
	c.363_364insGG;p.Ala122Glyfs*37	1	LP/P	Frameshift
	c.364_365insGG;p.Ala122Glyfs*38	1	LP/P	Frameshift
	c.381del;p.Leu128Phefs*31	1	P/P	Frameshift
	c.383T>G;p.Leu128Arg	1	P/P	Missense
	c.392A>C;p.Asn131Thr	1	P/P	Missense
	c.394C>T;p.Gln132*	2	P/P	Nonsense
	c.406_415del;p.Phe136Leufs*20	1	P/P	Frameshift
	c.406_407insGAT;p.Phe136*	1	LP/P	Nonsense
	c.414A>G;p.Pro138=	1	P/P	Splicing
	c.426_427insT;p.Asp143*	1	LP/P	Nonsense
	c.430G>T;p.Gly144*	1	P/P	Nonsense
	c.446C>A;p.Ala149Asp	1	LP/LP	Missense
	c.458T>C;p.Leu153Pro	1	LP/LP	Missense
Intron 2	c.463+2T>G	1	LP/LP	Splicing
	c.464-1G>C	1	P/P	Splicing
Exon 3	c.472C>G;p.Leu158Val	1	P/P	Missense
	c.473T>C;p.Leu158Pro	2	P/P	Missense
	c.481C>T;p.Arg161*	7	P/P	Nonsense
	c.481C>G;p.Arg161Gln	1	P/P	Missense
	c.482G>A;p.Arg161Gln	1	P/P	Missense
	c.486C>G;p.Cys162Trp	1	P/P	Missense
	c.486C>A;p.Cys162*	1	P/P	Nonsense
	c.488T>C;p.Leu163Pro	2	P/P	Missense
	c.490C>T;p.Gln164*	2	P/P	Nonsense
	c.499C>T;p.Arg167Trp	6	P/P	Missense
	c.500G>A;p.Arg167Gln	10	P/P	Missense
	c.506T>C;p.Leu169Pro	2	P/P	Missense
	c.523T>G;p.Tyr175Asp	2	P/P	Missense
	c.524dup;p.Tyr175*	1	P/P	Nonsense
	c.533T>C;p.Leu178Pro	1	P/P	Missense
	c.547_549dup;p.Ser183dup	1	P/P	IDI
	c.551T>C;p.Leu184Pro	1	P/P	Missense
	c.556G>T;p.Glu186*	1	P/P	Nonsense
	c.563T>G;p.Leu188Arg	1	P/P	Missense
	c.608_609del;p.Gln203Argfs*52	1	P/P	Frameshift
	c.629G>A;p.Arg210Glu	1	VUS/VUS	Missense
	c.640T>A;p.*214Argext*14	1	P/LP	Frameshift

Legend: IDI—in-frame deletion (insertion), P—pathogenic, LP—likely pathogenic, and VUS—variant of uncertain significance.

## Data Availability

The original contributions presented in this study are included in the article/Supplementary Materials. Further inquiries can be directed to the corresponding author.

## Supplementary Materials

The following supporting information can be downloaded at https://www.mdpi.com/article/10.3390/ijms27198804/s1. References [45,46,47,48,49,50] are cited in Supplementary Materials.

## Abbreviations

The following abbreviations are used in this manuscript:

ACMG	American College of Medical Genetics and Genomics
CanVIG	Cancer Variant Interpretation Group
CI	Confidence Interval
CCRC	Clear-Cell Renal Cancer
CNV	Copy Number Variation
ELOC	Elongin C
DNA	Deoxyribonucleic Acid
dNTP	Deoxyribonucleic Triphosphate
EDTA	Sodium Ethylenediamine Tetraacetate
HIF	Hypoxia-Inducible Factor
HGVS	Human Genome Variation Society
GoF	Gain-of-Function Variant
LoF	Loss-of-Function Variant
MLPA	Multiplex Ligation-Dependent Probe Amplification
MRI	Magnetic Resonance Imaging
NCCN	National Comprehensive Cancer Network
NMD	Nonsense-Mediated mRNA Decay
OMIM	Online Mendelian Inheritance in Man
PCR	Polymerase Chain Reaction
P/LP	Pathogenic/Likely Pathogenic variant
pVHL	VHL Protein
VHLS	von Hippel–Lindau syndrome
VUS	Variant of Uncertain Significance
USA	United States of America
