# Supplementary material for: Clinical and Genetic Characterization of Russian Patients with von Hippel–Lindau Syndrome
Source: Int J Mol Sci. 2026 Oct 1;27(19):8804. doi: 10.3390/ijms27198804 (PMC13665197; doi:10.3390/ijms27198804)
Supplement: Supplementary file 1 [file ijms-27-08804-s001.zip › ijms-4556913-supplementary.pdf]

Surveillance and treatment options in patients with VHLS

After a causative VHL variant is diagnosed in patient, a surveillance regimen is recommended at post-test genetic counseling. Currently, the most substantiated consensus could be considered is the consensus of the International VHLS Consortium and the patient-physician association "VHL-Alliance" in the USA, which takes into account a large number of original studies and the manifestation characteristics of different types of VHLS [1]. In brief, from the age of 1-2 years, examinations begin to detect retinal angiomatosis (hemangioblastomas) and pheochromocytoma using non-invasive methods; the concentration of metanephrines is also analyzed from the age of 5. An audiogram to exclude tumor of the inner ear and an MRI to detect CNS hemangioblastomas begin at the age of 11; MRI to detect VHLS, pheochromocytoma, renal and pancreatic cysts, and pancreatic tumors is performed starting at age 15. Physical and laboratory tests are performed annually, and MRI is performed every two years. However, the frequency of MRI of specific organs may be increased after disease manifestation and/or a prevalent accumulation of certain pathological manifestations of the syndrome in a family with VHLS. The age at which screening begins may be lowered at the discretion of specialist physicians. Generally, specialized screening for patients with VHLS is discontinued upon age 65 because dynamic screening has become routine, considering the oncological alertness of older adults and their cancer history.

A diagnosis of VHLS is important not only for determining a monitoring regimen but also directly influences treatment decisions. According to the National Comprehensive Cancer Network (NCCN) guidelines, CCRC resection in VHLS patient should be performed understanding the high risk of recurrent tumors in both kidneys and the preservation of functional renal parenchyma for as long as possible. Therefore, the "3 cm rule" is used: tumors are removed when they reach 3 cm in greatest dimension during limited resections, avoiding nephrectomy. However, the decision about the surgical intervention depends from the stage of CCRC, tumor invasion of major vessels, urine collecting system, and the risk of metastasis [2]. This approach is also used by Russian specialists in the treatment of patients with VHLS [3].

Treatment of multiple (metastatic) tumors in patients with VHLS including the immune checkpoint drugs and multikinase inhibitors, in particular pazopanib, remains relevant [2, 4]. At the same time belzutifan is a selective inhibitor of HIF2α – a molecule plays an important role in the HIF-mediated mechanism of carcinogenesis – currently recommended for the treatment of generalized forms of cancer in patients with VHLS. The indication for belzutifan is genetically confirmed VHLS and the presence of tumors that do not require immediate surgical removal: CCRC, CNS hemangioblastomas, and/or pancreatic neuroendocrine tumors. In some cases of systemic VHLS therapy, especially in multiple progressive hemangioblastomas, belzutifan is the only effective targeted drug with a pathogenetic mechanism of action and approved by the Food and Drug Administration (FDA) in the United States and in the European Union [5, 6].

1. Daniels A.B., Tirosh A., Huntoon K., Mehta G.U., Spiess P.E., Friedman D.L., Waguespack S.G., Kilkelly J.E., Rednam S., Pruthi S., Jonasch E.A., Baum L., Chahoud J., International VHL Surveillance Guidelines Consortium. Guidelines for surveillance of patients with von Hippel-Lindau disease: consensus statement of the International VHL Surveillance Guidelines Consortium and VHL Alliance. Cancer. 2023, 129(19), 2927-2940.

2. NCCN Clinical Practice Guidelines in Oncology. Kidney Cancer. v.2.2026. (https://www.nccn.org/guidelines/category_1., access date 15 June 2026).

3. Volkova M.I., Turupaev K.A., Filippova M.G., Ladyko D.D., Sinitsyna O.R., Gridneva Y.V., Matveev V.B. Renal cell carcinoma associated with von Hippel–Lindau syndrome: an ambispective study. Journal of Modern Oncology. 2025, 27(2), 122–128 [Russian].

4. Rinaldi L., Senatore E., Feliciello S., Chiuso F., Insabato L., Feliciello A. Kidney cancer: from tumor biology to innovative therapeutics. Biochim Biophys Acta Rev Cancer. 2025, 1880(1), 189240.

5. Park J., Arshad H., Khan S., Vinod N.P., Goel R., Vats D., Arshad I. Belzutifan in von Hippel-Lindau syndrome and renal cell carcinoma: mechanisms, clinical evidence, and future directions. Cureus. 2026, 18(5), e108967.

6. Baron A., Broekman E., van Gils M., Poplavska E., Mol P., Saliete I., Sepodes B., Pean E. The European medicines agency review of belzutifan (Welireg) for the treatment of adult patients with von Hippel-Lindau disease-associated tumors. Oncologist. 2026, 31(2), oyaf433.
